# Managing diabetes mellitus with comorbidities in primary healthcare facilities in urban settings: a qualitative study among physicians in Odisha, India

**DOI:** 10.1186/s12875-021-01454-4

**Published:** 2021-05-22

**Authors:** Sandipana Pati, Sanghamitra Pati, Marjan van den Akker, F. G. Schellevis, Krushna Chandra Sahoo, Jako S. Burgers

**Affiliations:** 1 Centre for Chronic Conditions and Injuries, Public Health Foundation of India, New Delhi, India; 2 Indian Institute of Public Health Bhubaneswar (PHFI), Plot No. 267/3408, Jaydev Vihar, Mayfair Lagoon Road, Bhubaneswar-751013, Bhubaneswar, Odisha India; 3 Regional Medical Research Centre, Indian Council of Medical Research, Bhubaneswar, Odisha India; 4 Institute of General Practice, Johann Wolfgang Goethe University, Frankfurt am Main, Germany; 5 Department of Family Medicine, Maastricht University, Maastricht, the Netherlands; 6 Academic Centre of General Practice, KU Leuven, Leuven, Belgium; 7 Department of General Practice and Elderly Care Medicine, Amsterdam Public Health Research Institute, Amsterdam University Medical Centers Location VUmc, Amsterdam, Netherlands; 8 NIVEL (Netherlands Institute for Health Services Research, Utrecht, the Netherlands; 9 Department of Family Medicine, School CAPRI, Maastricht University, Maastricht, the Netherlands; 10 Dutch College of General Practitioners, Utrecht, The Netherlands

**Keywords:** Diabetes mellitus, Comorbidity, Primary care physicians

## Abstract

**Aim:**

To explore the perceived barriers and facilitators in the management of the patients having diabetes with comorbidities by primary care physicians.

**Methods:**

A qualitative In-Depth Interview study was conducted among the primary care physicians at seventeen urban primary health care centres at Bhubaneswar city of Odisha, India. The digitally recorded interviews were transcribed verbatim and translated into English. The data were analysed using thematic analysis.

**Results:**

Barriers related to physicians, patients and health system were identified. Physicians felt lack of necessary knowledge and skills, communication skills and overburdening due to multiple responsibilities to be major barriers to quality care. Patients’ attitude and beliefs along with socio-economic status played an important role in treatment adherence and in the management of their disease conditions. Poor infrastructure, irregular medicine supply, and shortage of skilled allied health professionals were also found to be barriers to optimal care delivery, as was the lack of electronic medical records and personal treatment records.

**Conclusion:**

Comprehensive guidelines with on the job training for capacity building of the physicians and creation of multidisciplinary teams at primary care level for a more holistic approach towards management of diabetes with comorbidities could be the way forward to optimal delivery of care.

## Background

Optimal care of patients with diabetes mellitus continues to be a challenge to health systems around the globe. [1] Given the chronic and complex nature of the disease, the presence of additional comorbidities further multiplies this challenge. Prior studies have indicated the high prevalence of comorbidities in patients with diabetes, and the difficulties in managing these multiple conditions [2–4]. The problem is expected to be more complex in resource limited low- and middle-income countries (LMICs), where health systems are faced with a dual burden of infectious diseases and rise in non-communicable chronic conditions [5]. With inadequate and uneven distribution of specialized care facilities, most patients with diabetes in LMICs depend on an already overburdened primary care for their health care needs [6–8]. Furthermore, the traditional orientation of health systems towards infectious disease management, limited resources and fragmented primary care, pose an uphill task for the primary care physicians in managing patients with diabetes and comorbidities. Thus, it is imperative to understand the challenges faced by primary care health providers in LMICs settings towards the management of patients with diabetes and comorbidities.

As a central figure in disease management at primary care level, studies in the past have stressed the importance of primary care physicians’ role in quality management of patients with diabetes [9–11]. Alberti et al. in their primary care study conducted in Tunisia found that physician related factors like motivation and workload of the doctors, significantly affected diabetes care [12]. Abdulhadi et al. in their study in primary care in Oman found that trust deficiency on the competencies of allied healthcare staff and lack of teamwork approach are major barriers to quality diabetes care [13]. Venkataraman et al. explored challenges in diabetes management in India and also identified healthcare provider issues like lack of adequate knowledge, emphasis on acute management rather than preventive care, delay in clinical response to poor control and competing care demands as significant barriers to deliver quality care to diabetics [14]. Lall et al. in their study conducted in a rural district of India on challenges in primary care for diabetes and hypertension management have found fragmented care and a poor health information system as major barriers faced by physicians in quality management [15].

The public health care system in India has a three-tier structure comprising of primary, secondary and tertiary levels. Tertiary health care is provided by medical college hospitals, and the district and sub-divisional hospitals render secondary care. The primary healthcare centres are involved in delivering primary care. The role of primary care physicians is to provide outpatient clinical services. [16] With rapid urbanization and an increasing urban population, urban health has emerged as one of India's most important health concepts of the decade. The growing proportion of urban poor and disadvantaged having comparably lower health indices than rural areas face multiple social and financial barriers to quality healthcare. While urban health has been highlighted over the years, there has been little concerted effort at national level to provide urban populations with comprehensive health care.

Recently, primary health care in urban areas has been reinforced under the National Urban Health Mission (NUHM) scheme in 2013. [17] Urban primary health care centres (UPHCs) have been established specially to strengthen health status of urban poor, but there is limited information on primary health care among urban populations in India. According to the National Sample Survey Office’s 71st round on social consumption of health, about 54% of outpatient care in urban Odisha is provided by public healthcare facilities. [18] There is a lack of perspective among health care providers on the management of patients with diabetes and comorbidities among urban populations in India. In order to develop effective policies and interventions, it is imperative to understand the challenges and opportunities of managing diabetes and the comorbidities of the local population. However, these have not been addressed among urban populations in India. The present study therefore explored the perceived barriers and facilitators of primary care physicians in the management of diabetes and comorbidity patients in urban settings.

## Methods

### Study design, setting, and participants

A qualitative In-Depth Interview (IDI) study was conducted among the primary care physicians at all seventeen governmental UPHC, under the Capital Hospital at Bhubaneswar, the capital city of Odisha, India.

According to the 2011 census, Odisha had 42 million inhabitants, of whom about 17 per cent lived in urban areas – around one-fourth were slum dwellers, those who depended on the public health system for primary health care. Looking at the trends in urbanization, the district of Khurda has the highest rate of urbanization in 2011 with 48%, while the majority of the urban population in the district of Khurda resides in Bhubaneswar. [19]

For the present study, the primary care physicians involved in treating patients in the respective UPHCs were interviewed. A single doctor is appointed for each UPHC. We selected IDI for data collection as the number of study participants was limited and it was also not feasible to organize them for the focus group discussion. Among the participants, ten were female and seven male; with an average age of 40 (range 28 to 61). The number of years of experience as primary care health provider ranged from 1.5 to 25 years (average 12 years). Among them three participants had received a specific training in managing diabetes mellitus. All participants were contacted in person by the first author (SP1) before the interviews. All participants participated voluntarily; no payment was offered nor given.

### Data collection procedure

The data were collected using an In-depth interview guide Table 1. The interview guide was developed by the first and second author, based on findings from previous studies. [11, 20, 21] All interviews were conducted in local language (Odia) and English. The interview lasted for an average of 30 (range 20–50) minutes. The interviews were conducted at the UPHCs by two trained interviewers who have an educational background in public health and qualitative research as well as command of Odia and English language. In this study all the authors are from various educational and professional backgrounds; the diverse educational background and nationalities of all the authors brought their unique perspective and enhanced the conformability of the study findings.

**Table 1:** In- Depth Interview (IDI) guide

Self-introduction of the participant
Probe: sociodemographic and professional details
Could you describe about your health facility?
Probe: staff, location, funding/budget, patients/day, functioning hours, drugs and investigations availability, connectedness with specialized facility
How do you manage diabetes and diabetes with comorbidity?
Probe: patient load, out-patients care, chronic diseases, comorbidity, complications in comorbidity and care-practice
How do you try to redress these problems?
Probe: seek further information, sources, consultation with specialists -how often, continuity of care
What are considered as good outcomes for patients receiving care for diabetes comorbidity?
Could you tell in detail about patients who might find it difficult to cope well with diabetes comorbidity?
Probe: problem they faced, coping strategies, and any initiative or suggestion
In your opinion what is the role of physicians in supporting those not currently coping well with diabetes comorbidity?
Probe: role of other members of the primary care facility like nurse, pharmacist and others
In your view what would help you to help patients to cope better with diabetes comorbidity?
Probe: training/support, patient support, techniques, skills, and mentoring mechanism
What kind of additional support do you feel you need to deal with such patients?
Probe: staff, guidelines, referral system

### Data management and analysis

The digitally recorded interviews were transcribed verbatim and translated into English. The data were analysed using thematic analysis. [22] In the process of transcribing and translating the data we familiarized ourselves with the data before stating the coding and identifying themes; then we coded the data. After coding we identified codes – categories – themes. The emerged themes were reviewed and finalize. After primary check of the results by the third author, consensus was reached through discussions with the other researchers, who are from public health background and experienced in qualitative research. In order to avoid any misinterpretation, during the coding of the data, both Odia and English versions of the transcripts and in some complex cases, digital data were simultaneously used.

### Ethical considerations

The Odisha state research and ethics committee gave the ethical approval for the study (letter no. 161/SHRMU dt. 16.05.2014). All participants were informed about the purpose of the study and consent was obtained from them. The identities of the respondents were kept confidential.

## Results

Three major themes emerged: 1) Health system preparedness to manage diabetes comorbidities, 2) Challenges faced by physician to treat diabetes comorbidities, and 3) Patients' related factors in management of diabetes comorbidities. (Table 2) The findings are presented under each theme and category with quotes from the participants.

**Table 2:** Major themes, categories and codes

Theme	Categories	Codes
Health system preparedness to manage diabetes comorbidities	Challenges in relation to infrastructure and logistics	Supply of medicine Laboratory facilities Inadequate human resources Lack of trained support staff
	Management of records and documentation	Poor record keeping Improper registration of chronic disease patients Strengthen formal referral channels Support of pharmaceutical companies
	Community participation and literacy	Awareness campaigns among community members Active engagement of community health workers
Challenges faced by physician to treat diabetes comorbidities	Barriers to provide effective treatment	Lack of confidence Lack of orientation and training on skill development High patient density
	Facilitators for effective treatment	Skill development on time management Networking with seniors, specialists Patient physician relationship
Patients' related factors in management of diabetes comorbidities	Treatment adherence	Multiple issues affecting the adherence Non-compliance Traditional beliefs Attitudes towards change and appraisal of comorbidity
	Healthcare expenditure	Socio-economic status Out-of-pocket expenditure Incentives, Subsidy, Insurance
	Maintaining a personal treatment record	Previous prescription Care-seeking pathway history Diagnostic reports

### Theme 1: Health system preparedness to manage diabetes comorbidities

The health system played a crucial role in the management of patients. While lack of regular supply of medicines, poor laboratory services in the health facility, lack of trained human resources were some of the common barriers for all physicians, few facilitators like NCD awareness campaigns and periodic follow up of the patients by the community level health workers helped in improving the quality of management.

#### Category 1: Challenges in relation to infrastructure and logistics

Medicines: The dependence of UPHCs on the government’s central medicine store for their medicines frequently leads to irregular supply of medicines to the UPHCs for NCDs like diabetes mellitus and hypertension. Moreover, the unavailability of medicines for different chronic conditions was also considered to be an important barrier by all physicians.

*P4- “To the only diabetic patient we give the medicines to decrease the blood sugar level but in comorbidity we also have to treat the other diseases and sometimes we don’t have the supply of diabetes medicines... in other chronic diseases we have for acid peptic disease… blood pressure also the medicines come but periodically”.*

Laboratory facilities: All physicians felt that the absence of good laboratory facilities hampered their management of diabetics with comorbidities. The dependence on reports from private laboratories which were either unreliable or proved to be a cost burden for the patients forced them to refer the patients to higher level of public health care facilities.

*P4- “Patients want all tests to be done at one place but due to unavailability of these facility they have to face many problems.”*

Inadequate human resources and lack of trained support staff: Most of the facilities were affected with lack of skilled staff and in many cases the allied health personnel were handling more than one responsibilities. This hampered delivering multidisciplinary services necessary for diabetes patients with comorbidity.

*P10- “without proper staff it’s difficult to manage a UPHC... Now we are 3 staffs here… I doctor, one pharmacist and one sweeper…. we don’t have any attendant or ANM or staff nurse. If both of them are missing in a same day I face a lot of problems managing alone”.*

#### Category 2: Management of records and documentation

Poor record keeping or no registration of chronic disease patients: The absence of any formal record or shared record of chronic disease patients made the task of management and multidisciplinary collaboration difficult.

*P9- “They come with the reports after tests and with no record we don’t know their condition in the past and they do many tests again and again. Repetition mostly happens because we don’t keep any records”.*

Lack of formal referral and back referral channels: On referral procedures, participants shared that they there is no formal record system of referral and back referral and most of the time they do not know the outcome of their referral to higher centres. They felt that a back referral record or feedback would make them more connected and involved with the treatment of the conditions of the patient.

*P1- “I send the complicated cases to the Capital hospital… sometimes they come back with reports… sometimes they don’t come”.*

Pharmaceutical companies: The support by pharmaceutical companies and medicine representatives in provision of journals and updates on the new treatment and drug composition was perceived as an enabler by some participants. They felt it helped them in staying abreast of the latest treatment modalities in chronic conditions.

*P16- “Pharma companies also give us a lot of journals for diabetes and other chronic diseases… and medicine representatives inform about new medicines and their composition”.*

#### Category 3: Community participation and literacy

Awareness campaigns: The awareness campaigns that included information, education and communication (IEC) on NCDs in the community and health facilities was perceived as a facilitator to management. It was felt these activities encouraged patients to seek advice for their conditions and made them more attentive to their treatment.

Community level health workers: The role of community level health workers like Multi-Purpose Health Workers (MPHW): The role of MPHW in creating awareness among population and following up with the patients personally on their treatment and health conditions helped the physicians to be more responsive in assessing the patients in the health facility catchment area. They also brought the more serious patients with multiple conditions to their notice for intensified care.

### Themes 2: Challenges faced by physician to treat diabetes comorbidities

#### Category 1: Barriers to provide effective treatment

Barriers in relation to knowledge and skills: The lack of formal training on diabetes and comorbidities management was perceived as a major constraint for quality management of diabetes patients with comorbidities. Physicians expressed that managing such patients especially those on insulin treatment was beyond their knowledge domain and they did not have the necessary clinical skills to manage, hence they referred them to a specialist. Most relied on internet, journals, books and sometimes representatives from pharmaceutical companies to update themselves. Those physicians who had not received any training were not aware of standard clinical guidelines for treatment of chronic conditions.

*P10- “they give training only on the current epidemic… yes of course, if there would have been some training on NCD…then there we could have a standardised procedure for treatment, we could have followed a protocol of state govt. By which we could sort out the problems we are facing daily”.*

Barrier in relation to communication skill: Physicians felt they were unable to put forth the necessary advice to the patients with diabetes and comorbidities in an effective way and agreed that communication skills could help in overcoming this barrier. They opined the specialist or endocrinologist can help the patient to understand his comorbid condition better and therefore they referred the patient to a specialist.

Patient load: The average time spent by the physicians for a consultation with a diabetes patient ranged from three to ten minutes. They expressed that within limited working hours and overburden of patients it is difficult to reserve adequate consultation time and it affected the quality of care. Most UPHCs in the study were managed by one doctor, and daily patient attendance ranged from 50 to 150.

*P10- – “that depends if rush of patients is there then I don’t give much time. If few patients are there I give them minimum 5 min…. Some patient come with 3–4 last test reports in the rush time… I get irritated …still I treat them”.*

#### Category 2: Facilitators for effective treatment

Facilitators for knowledge and skills: A limited number of participants had a formal one-year training in diabetes mellitus expressed confidence in handling the diabetes patients with comorbidities. Similarly, physicians who had attended any seminar or workshop on non-communicable diseases (NCDs) were more optimistic on handling of these cases. They were aware of the standard clinical guidelines for chronic conditions management and treatment protocols.

*P3- “I have done a one-year diploma course in diabetes management…so I have no problem as such in clinical treatment”.*

Facilitators for communication skills: The physicians who were confident in communication with the diabetes patients on their disease conditions found their patients to be compliant to the advices and more involved in the treatment plan. It was observed that those physicians who had received diabetes management training did not have any difficulty in communicating with the patients.

Time management skill: Physicians trained in diabetes management had allocated a separate day in a week for diabetes patients and expressed satisfaction about the time (about 20 min) devoted for consultation of these patients.

*P6- “If a diabetes patient comes in a rush time who needs more time from me, I advise the patient to come on our weekly diabetes day... they have other problems also like heart problem, neuro, kidney related problem, I can spend more time with them by explaining them about how to change their lifestyle. I also advise on exercise, diet. It crosses 20- 30 minutes sometimes”.*

Networking with seniors, specialists: Physicians who discussed and sought advice from senior colleagues and specialists in higher centres felt more confident in handling the diabetic patients. Few physicians who went once a week to a centre with specialists for duty, observed the specialists, senior colleagues’ management practices (peer learning) and felt more confident in handling the patients in their own facility.

In the management of diabetics with comorbidities there were factors relating to physicians, which were barriers and facilitators to quality care. Those physicians who had had any training in diabetes kept a separate day for managing diabetes patients and did not perceive the care for diabetes patients with comorbidities as added burden, while others who were not trained felt overburdened when treating them in their daily practice. It was observed that training correlated to increased confidence and time management and the trained physicians appreciated the additional needs of patients with diabetes and comorbidities. The trained physicians did not express any difficulty in communicating with diabetes patients about comorbidities but others found it hard to communicate.

Empathy: Counselling and maintaining an empathetic relationship with the patients was found to be a facilitator. All physicians agreed on the importance of patient education and counselling. Physicians who had an empathetic relationship with the patients and counselled them felt they had cooperative and satisfied patients. They also felt they were able to manage the multiple demands of patients with diabetes and comorbidities.

*P3- “I treat them as my family member. I try to make them understand everything clearly … So might be for this they follow my advice and are regularly coming to me”.*

### Theme 3: Patients' related factors in management of diabetes comorbidities

Majority of the patients seeking care from the primary urban health center belong to slums or belong to low socioeconomic group, according to the physicians – most of them have poor financial conditions, low or moderate literacy and migrants. Physicians felt that patient’s cooperation, financial condition and awareness played a significant part in the management of diabetes. While a non-compliant patient or apprehensive patient’s adherence to treatment was poor, a cooperative patient made management easy. Similarly, the economic background dictated many of the treatment choices during management.

#### Category 1: Treatment adherence

Physicians felt diabetes patients’ adherence to the treatment to be an important factor in quality management. Patients who adhered to treatment had better clinical outcomes and less referrals than the non-adherent patients with diabetes. They expressed multiple issues affecting the adherence.

Attitude and belief: Physicians reported occasionally that patients discontinued their treatment with the assumption that they were cured. Later deterioration of the conditions led to more challenges in management for the physician. Mistrust of patients on quality of care supplied at the health care facilities also influenced the adherence to prescribed treatment.

*P7- “some have the affordability but they are not serious……they get irritated for having to take medicine regularly”*

*P15- “Some people are health conscious…they ask questions…. what to eat…complications of diabetes…they also do regular follow up”.*

Appraisal of comorbidity: Physicians felt some patients perceived their multiple chronic conditions and treatment as non-serious and did not warrant regular treatment, which decreased their adherence to treatment and follow up.

*P2- “Some people hesitate to take those drugs……. those few which is in supply. They have wrong notion towards government supplied medicines that they are not good”.*

#### Category 2: Healthcare expenditure

Low economic status of the patients was found to be a barrier to management as low affordability of patients affected the choice of drug therapy, giving priority to the most urgent condition only. Physicians felt frustrated when patients with poor financial power who need specialist care were hesitant to go to specialized centres upon referral and insisted on being treated at the primary care level.

*P16- “depending upon his financial condition and how much he can spend regarding medicines, as per that I prescribe medicine for the most necessary condition”*

*P9- “Most of the diabetes cases with many conditions we refer but some people do not prefer to go because of losing money”.*

#### Category 3: Maintaining a personal treatment record

Patients with diabetes who maintained a record of their past treatment were found to be more cooperative and aware about their health conditions by the physicians compared to those without a medical record. They perceived this to be helpful in avoiding duplication of tests and keeping a track on the treatment plan of comorbidities. Those patients with records were also more observant to deterioration of their conditions and sought prompt treatment.

## Discussion

The present study explored the perceived challenges faced by primary care physicians in managing diabetes patients with comorbidities at their health facilities. All primary care physicians noted that they found managing multiple chronic conditions challenging. Like in past studies our study participants also felt the lack of services like equipped laboratory, provision of medicines, trained support staff to be barriers to effective management. [23, 24]

We found that there was minimal provision of training and courses from the government for primary care physicians for chronic conditions management. Few physicians had undergone self-sponsored formal training, the rest used internet, books, and journals for their information. It was surprising that some participants depended on pharmaceutical companies and their representatives for their knowledge update on treatment modalities and considered it to be a reliable source of information. The reliance on pharmaceutical company representatives, however, is a matter of concern, as multiple studies in the past have recorded that in the absence of formal training undue influence of pharmaceutical companies in the treatment prescribing pattern among general practitioners can lead to suboptimal care with higher costs. [25, 26] It was seen that physicians with training had made time management innovations like assigning a special day for diabetes patients and communicated better with their patients and had greater patient compliance. This also indicates the strong relationship between effective communication and treatment compliance. Few studies in the past have also highlighted the importance of effective physician patient communication in quality diabetes care. [27] The findings of our study further underscore the need of formal and suitable training for the primary care physicians. There is also a strong aspect of health inequalities, for example, those diabetes patients with access to education can also afford better medicines and are more likely to keep records than poorer and less well-educated patients.

Another finding was the importance of a peer network for improved management. Participants who had access to specialists and endocrinologist felt they were able to handle the multiple conditions better. Similarly, participants felt encouraged and confident in managing patients with diabetes who had been back referred to them by specialists. These findings highlight the need for a robust knowledge and medical record sharing and hand holding network among the primary care physicians and specialists. Participants also felt that patient awareness, better appraisal of their disease condition, healthcare expenditure and adherence to treatment were interrelated. It was also seen that physicians found it helpful if the patient had maintained the past treatment records and this facilitated their management. However, in the absence of an electronic medical record system in their health facilities physicians had to rely on patients to maintain a self-record. The implementation of electronic medical records in primary care shall facilitate better management and avoid laboratory testing and treatment overlap. The provision of records could also help in identifying patients with more care needs and the community health workers can accordingly follow up with them.

Our study findings reinforce the need for comprehensive training in chronic conditions management at the primary care level. Though training has been a vital component of the National Health Mission (NHM), [28] it has been more focused on the Reproductive Maternal and Child Health. As studies already prove the considerable burden of diabetes and comorbidities at primary care level, a standardized training designed for appropriate management of multiple chronic conditions at primary healthcare level would be a welcome step.

As a part of Health and Wellness Centre (HWC) [29] initiative under Universal Health Coverage programme of government of India, a teamwork approach for chronic conditions management may be considered. Mental health counsellors under Mental Health Programme [30] and physiotherapists under geriatric clinics that are intended to be placed at primary care level under HWC initiative, maybe integrated with AYUSH (Ayurveda, Yoga and Naturopathy, Unani, Siddha, Homeopathy) [31] component of NHM and National Programme for Prevention and Control of Cancer, Diabetes, Cardiovascular disease and Stroke [32] at primary care level to create a multidisciplinary team to facilitate all-inclusive management of patients with multiple chronic conditions.

Though computers are present in the UPHCs, their usage has been limited to administrative work and report sharing. The information and communication technology may be used in maintaining a database of chronic condition patients with regular updating and follow up. Maintenance of electronic health records could be a solution for a standardized record.

The strength of our study is the representation of participants from all UHPCs of Bhubaneswar at the time of the data collection. However, there has been an increase in the number of UPHCs in Bhubaneswar since then. The participants were both male and female, which is another strength. Rigorous qualitative methods were applied and accordingly the findings were discussed with some of the participants after the data analysis. Though in a qualitative study generalisability of the findings is not the aim, our study findings being comparable to past studies improve the plausibility and transferability of our findings in similar settings. Since no quantitative conclusions are possible, the findings need to be confirmed in larger study.

In conclusion, amongst the multiple barriers as perceived by the physicians like inadequate communication skills, poor patient compliance and shortage of allied health professionals, the lack of training is seen as the most important barrier to effective management. As training is seen to impact other aspects of management like counselling and time management, it is recommended that primary care physicians receive regular trainings and capacity building exercises on the multiple chronic conditions management along with communication skill development training for optimal care delivery. Our study findings on the perceived barriers and facilitators may further be applied in larger quantitative study to identify the more dominant barriers and facilitators to quality care and appropriate interventions can be designed accordingly.

## Conclusion

With the growing burden of diabetes patients with comorbidities, a robust primary care is the need of the hour. It is imperative that to meet the complex needs of diabetes patients a primary care physician needs to be trained, not just in the curative aspect but also in communication and counselling and is assisted with a coordinated team of multidisciplinary professionals. Effective management of diabetes patients with multiple comorbidities calls for a holistic approach with well-equipped and strengthened primary care.

## Data Availability

The data is available with the corresponding author, and can be made available on reasonable request and permission from State human resources management unit, Department of Health and Family Welfare, government of Odisha.

## Abbreviations

LMIC: Low and Middle Income Countries
IDI: In-Depth Interview
UPHC: Urban Primary Healthcare Centre
NCD: Non-Communicable Disease
IEC: Information, Education and Communication
MPHW: Multi Purpose Health Worker
NHM: National Health Mission
HWC: Health and Wellness Centre
AYUSH: Ayurveda, Yoga, and Naturopathy, Unani, Siddha, Homeopathy
